# Enhanced Enrollment in the National Diabetes Prevention Program to Increase Engagement and Weight Loss for the Underserved: Protocol for a Randomized Controlled Trial

**DOI:** 10.2196/15499

**Published:** 2020-06-01

**Authors:** Natalie D Ritchie, Jodi Summers Holtrop, R Mark Gritz, Katherine Ann Sauder, Michael Josh Durfee, L Miriam Dickinson, Peter G Kaufmann

**Affiliations:** 1 Office of Research Denver Health Denver, CO United States; 2 Department of Psychiatry University of Colorado School of Medicine Aurora, CO United States; 3 University of Colorado College of Nursing Aurora, CO United States; 4 Department of Family Medicine University of Colorado School of Medicine Aurora, CO United States; 5 Adult and Child Consortium for Health Outcomes Research and Delivery Science University of Colorado School of Medicine Aurora, CO United States; 6 Division of Health Care Policy and Research University of Colorado School of Medicine Aurora, CO United States; 7 Lifecourse Epidemiology of Adiposity and Diabetes Center University of Colorado School of Medicine Aurora, CO United States; 8 Department of Biostatistics and Informatics Colorado School of Public Health Aurora, CO United States; 9 Villanova University College of Nursing Philadelphia, PA United States

**Keywords:** type 2 diabetes, prevention, weight loss, diabetes mellitus, type 2, cohort studies, self efficacy, vulnerable populations

## Abstract

**Background:**

Type 2 diabetes affects 9.4% of US adults with higher rates among racial and ethnic minorities and individuals of low socioeconomic status. The National Diabetes Prevention Program (NDPP) is an evidence-based and widely disseminated behavioral intervention to reduce diabetes incidence through modest weight loss. However, retention in the yearlong NDPP is problematic and leads to suboptimal weight loss, especially among diverse, underserved populations. Strategies to improve NDPP engagement and weight loss are needed urgently. Pilot results of the pre-NDPP, a novel enhancement to enrollment in the NDPP based on the Health Belief Model, were highly successful in a nonrandomized cohort study among 1140 racially diverse, predominately low-income participants. A total of 75 presession participants had doubled attendance and weight loss as compared with earlier participants who did not receive presessions. On the basis of these promising results, we are conducting a randomized controlled trial (RCT) to determine whether pre-NDPP reliably improves NDPP outcomes, as reported on ClinicalTrials.gov.

**Objective:**

This study aims to (1) conduct an RCT comparing NDPP attendance and weight loss outcomes between participants who receive pre-NDPP versus direct enrollment into the NDPP (usual care), (2) examine potential effect mediators (perceived risk for developing diabetes and self-efficacy and readiness for weight control) and moderators (race and ethnicity; income level), and (3) evaluate implementation factors, including cost and projected return on investment.

**Methods:**

This two-arm RCT will compare outcomes among diverse, predominately low-income participants who receive pre-NDPP versus direct enrollment into the NDPP (usual care). This is a type 1 hybrid effectiveness-implementation design to determine clinical effectiveness through an RCT, while assessing factors that may impact future pre-NDPP dissemination and implementation, including cost. Our primary research question is whether pre-NDPP improves NDPP attendance and weight loss compared with standard NDPP delivery.

**Results:**

This project was funded in April 2019. Recruitment is underway as of July 2019. Initial participants began the intervention in October 2019. Data analysis and results reporting are expected to be completed in 2024.

**Conclusions:**

This RCT of pre-NDPP may lead to future dissemination of a scalable, evidence-based strategy to improve success of the NDPP, reduce disparities in NDPP effectiveness, and help prevent type 2 diabetes across the country.

**Trial Registration:**

ClinicalTrials.gov NCT04022499; https://clinicaltrials.gov/ct2/show/NCT04022499.

**International Registered Report Identifier (IRRID):**

PRR1-10.2196/15499

## Introduction

### Background

Type 2 diabetes (T2D) affects 9.4% of US adults with higher rates among racial and ethnic minorities and individuals of low socioeconomic status [1]. The Diabetes Prevention Program was a successful clinical trial demonstrating that intensive lifestyle support for weight loss initially reduced diabetes incidence by 58% [2]. The intervention was translated into the National Diabetes Prevention Program (NDPP) and disseminated by the Centers for Disease Control and Prevention (CDC) as a yearlong group-based program since 2012 [3]. Despite successes, a 2017 report found that retention in the NDPP is problematic and leads to suboptimal weight loss [4]. Attendance and weight loss are especially low among Hispanic, non-Hispanic black (NHB), and low-income non-Hispanic white (NHW) participants [4-6]. Strategies to improve NDPP outcomes among these disadvantaged populations are urgently needed.

We previously developed and pilot tested an NDPP enrollment protocol, the pre-NDPP, that provides a *presession* with three main components: (1) education about diabetes risks, (2) motivational interviewing (MI) to encourage participation in the NDPP, and (3) problem-solving of barriers to engagement [7]. The Health Belief Model [8] is the theoretical model underlying pre-NDPP, which posits that perceived risk, benefits of, barriers to, and cues to action, and self-efficacy are factors determining health behavior. As such, interventions to prevent T2D should focus on increasing risk awareness and exploring pros and cons of available interventions, including the NDPP. Studies have demonstrated that increasing awareness of diabetes risks may lead to risk-reduction behavior [9,10]. MI [11] may further facilitate action-oriented decision-making about NDPP engagement with its empathic coaching style and evocation of *change talk*. Multiple systematic reviews support the intended use of MI in a brief, group-based intervention to improve NDPP effectiveness [12-15].

The presession enhancement showed highly successful results upon initial dissemination in a diverse, predominately low-income population and may be a viable strategy to address suboptimal NDPP outcomes [7]. In a longitudinal cohort study among a diverse and underserved population, outcomes of 75 pre-NDPP participants who enrolled in the NDPP were compared with 1065 prior participants using the analysis of covariance and multivariable logistic regression. Presession participants stayed in the NDPP 99.8 days longer (*P*<.001) and attended 14.3% more sessions (*P*<.001) on average than those without a presession. Presession participants lost 2.0% more weight (*P*<.001) and were 3.5 times more likely to achieve the 5% weight loss target (*P*<.001).

### Objective

As reported on ClinicalTrials.gov (ID NCT04022499), we are now conducting a large intent-to-treat (ITT) randomized controlled trial (RCT) to test effects of the pre-NDPP enhancement on NDPP attendance and weight loss among a diverse, predominately underserved population with elevated diabetes risks. The primary outcome is percent weight loss. Additional aims include the examination of mediators and moderators of pre-NDPP outcomes and evaluation of the implementation factors of pre-NDPP.

## Methods

### Overview

We are recruiting 500 participants at risk for developing T2D from a large safety net health care system. Eligible, consenting participants are randomized 1:1 to either the usual care control group who are enrolled directly into the NDPP or the intervention condition who receive a presession prior to the NDPP (pre-NDPP). NDPP classes are held separately for the intervention and control groups to prevent contamination of pre-NDPP effects. Outcomes are NDPP attendance and weight loss. New NDPP classes are staggered to commence quarterly over 2.5 years. In addition to measuring main outcomes and potential mediators and moderators of treatment effects, we are collecting data related to program implementation to inform future dissemination. This RCT includes pragmatic research elements to support comparison to current NDPP outcomes and facilitate future dissemination, including (1) recruiting diverse individuals who meet CDC-established eligibility criteria for the NDPP with no further exclusions, (2) identifying eligible individuals through referrals from health care providers according to best practices [16], (3) providing the NDPP to both treatment conditions without altering its core structure, apart from the inclusion of a presession prior to the first NDPP class (for the pre-NDPP arm), (4) implementation in health care settings in which the NDPP is routinely available, and (5) using standard measurement of NDPP outcomes. The Colorado Multiple Institutional Review Board (18-2542) approved study procedures, and all participants will provide written informed consent before enrollment.

### Setting

Denver Health (DH) is an academic medical center and integrated health care system that is nationally recognized for its model of care for the underserved. DH serves 1 in 4 residents of Denver, Colorado, with a patient population that is 50% Hispanic, 15% NHB, and 30% NHW. In addition to a tertiary-care hospital and trauma center, DH operates 10 primary care clinics across the region. DH was an early adopter of the NDPP and to date has launched 61 yearlong NDPP classes across its network of primary care clinics with over 1400 participants to date.

### Eligibility

As a pragmatic trial, we are recruiting English- and Spanish-speaking adults who meet CDC-established NDPP eligibility criteria, including BMI≥25 kg/m^2^ (≥23 kg/m^2^ if Asian) and history of recent prediabetes or former gestational diabetes mellitus (GDM) diagnosis [17]. Prediabetes is based on a laboratory test within the past year in the individual’s electronic health record (EHR) that indicates a fasting blood glucose of 100 to 125, blood glucose of 140 to 199 measured 2 hours after a 75 gm glucose load, or hemoglobin A_1c_ of 5.7 to 6.4. GDM is based on past diagnosis in the medical record or self-reported. Patients without known prediabetes or past GDM may also be eligible based on a risk-screening tool [18], as administered by Lifestyle Coaches during recruitment. Participants are excluded if pregnant at enrollment or known to have T2D.

### Recruitment

We are identifying potential participants through referrals from health care providers at DH, which is known to support initial enrollment in the NDPP [16]. Providers refer through the EHR per usual practice. We also identify participants from a risk registry based on EHR data as needed to meet recruitment goals. The enrollment process after initial identification is as follows: (1) Lifestyle Coaches contact referrals by phone to verify interest, eligibility, and schedule eligible individuals for an initial screening visit, (2) consenting individuals are randomized to receive pre-NDPP or usual care NDPP and (3) complete an initial assessment (behavioral and anthropometric assessments), (4) the pre-NDPP group completes a presession 1 to 2 weeks before NDPP classes start, (5) both groups complete a follow-up behavioral assessment 1 to 2 weeks before NDPP classes start (after presessions are completed for the pre-NDPP arm), and (6) both groups commence yearlong NDPP classes.

We will enroll 500 participants, with a goal of 400 randomized participants (allowing for 20% attrition) across both groups attending ≥1 NDPP session as based on statistical power estimates presented below. From previous experience, we expect approximately 50% of referred patients to express interest in participating upon initial outreach. Then, after initial assessment, we expect early attrition of approximately 20% of consenting participants. We seek to recruit 50 individuals every 3 months, for a total of 500 participants recruited over 2.5 years (ie, 200 participants annually). Demographic characteristics of individuals in this study are expected to approximately match characteristics of all previous NDPP participants at DH: 78.0% female, 58.2% Hispanic, 19.5% NHB, 21.0% NHW, 61.4% low income (including a majority of low-income individuals within each racial and ethnic group), and a mean age of 48.4 (SD 12.7) years.

### Randomization

We are randomizing eligible, consenting participants to receive either the enhanced intervention (pre-NDPP + standard NDPP) or usual care control group (standard NDPP only) in a 1:1 ratio. Specifically, at the conclusion of the initial recruitment visit, consenting participants receive a preprepared *randomization packet* that denotes their group assignment and relevant details (eg, all class dates and times, Lifestyle Coach contact information). Packets are prepared in advance in a random order using a random number generator and sealed, such that research staff and participants are blinded to condition until the time of assignment.

### Retention

For generalizability, we are delivering NDPP with only customary retention methods, including offering classes at clinics where participants receive their primary care, facilitating transportation (eg, providing free parking and information about insurance-provided transportation benefits), offering make-up sessions as needed, and updating participant contact information often. To accommodate additional data collection required of participants in both arms of the trial (above and beyond routine care in the NDPP), we are providing compensation of US $25 for completing each of two research assessments at the time of initial recruitment and immediately prior to attending the NDPP. We are also providing an additional US $25 for all participants to complete a final weight measurement at 12 months.

### Description of the National Diabetes Prevention Program (for Both Conditions)

The yearlong NDPP promotes modest weight loss through diet and physical activity. The curriculum is published by the CDC [3]. We follow the CDC guidelines for implementing the standard group-based NDPP [17], including 16 weekly to biweekly sessions, followed by ≥6 monthly sessions over a total of 1 year. The objective of NDPP is achieving ≥5% weight loss. Attending more sessions is associated with greater weight loss [4,19], and guidelines allow NDPP sites to offer more than the minimum 22 sessions to support this goal. We offer 25 total NDPP sessions (16 in months 1-6; 9 in months 7-12), held at the same time, day, and location in group visit rooms available at 6 to 8 neighborhood primary care clinics. Two new NDPP classes commence quarterly over 2.5 years. To minimize potential contamination, participants in the two study arms are enrolled in separate NDPP classes. Trained, bilingual lay health educators lead NDPP classes as Lifestyle Coaches and provide make-up sessions as needed. They are observed by the research coordinator for fidelity and to assess for potential bias in NDPP delivery. Weight is measured at each session on a high-capacity, medical-grade scale. As required by the NDPP curriculum, participants are encouraged to achieve a weekly goal of ≥150 min of moderate to vigorous intensity physical activity (beginning gradually as needed). Participants are instructed to track start and stop times and report total weekly activity minutes at the following session. The most recent CDC curriculum also encourages a low-fat diet but does not require monitoring of dietary adherence [3,17]. Lifestyle Coaches conduct support calls between sessions to support engagement and health behavior change, address individual questions and concerns, and remind participants about upcoming sessions.

### Description of the Pre-National Diabetes Prevention Program Protocol

The pre-NDPP protocol was previously developed in a pilot study funded by the Colorado Department of Public Health and Environment. The protocol is based on the Health Belief Model [8] and extensive stakeholder engagement, including feedback from previous NDPP participants and Lifestyle Coaches. Presessions are intended to increase motivation and readiness to engage in the NDPP, while helping participants become comfortable with the group class format. Content was developed for a fourth grade reading level. Presessions focus on the following: (1) education on diabetes risks, (2) MI to participate in the NDPP, and (3) problem solving of barriers to engagement, following a standardized intervention manual. Presessions are delivered in a group format and scheduled for 1 hour to minimize burden, but are flexible in practice, lasting 60 to 90 min to address participant questions and needs. Presessions are held 1 to 2 weeks before NDPP classes start, at the same day, time, and location to facilitate transitions to the NDPP. To minimize bias, Lifestyle Coaches are alternatingly assigned each quarter to deliver pre-NDPP versus usual care NDPP, with accompanying fidelity observations.

Pre-NDPP participants first receive education on diabetes risks and information about available resources to reduce risk, including a description of the NDPP. Education is informed by the Health Belief Model [8] in which perceived risk, severity, benefits of and barriers to action, and cues to action determine health behavior. Topics include (1) an overview of T2D (eg, prevalence and common complications) and risks for developing T2D (eg, prediabetes, sedentary lifestyle, and overweight and obesity), (2) rates of T2D onset, (3) guidance that modest weight loss can reduce risk, and (4) evidence-based resources to prevent T2D, including a detailed overview of the NDPP. Guidance is intended to normalize the experience of being at-risk for T2D to reduce anxiety, while focusing on instilling hope that T2D is preventable and making calls to action.

Following the pre-NDPP manual, coaches then use MI techniques (eg, reflective listening, evoking ambivalence, rolling with resistance, and eliciting change talk) [11] to help participants identify their preferred plan of action to reduce risk, encouraging participation in NDPP sessions. For example, to create discrepancy, coaches acknowledge the difficulty of making changes in health behavior and probe for typical experiences of weight loss followed by weight regain or other similar challenges. To counterbalance these challenges, coaches will encourage participants to describe why preventing T2D is important to them (eg, wanting to live a long and healthful life or setting a positive example for their children and grandchildren). Coaches also nonjudgmentally acknowledge that while the NDPP works well for those who attend regularly, it may be challenging for some individuals to attend a yearlong class, and that it is okay to opt out or choose other risk reduction resources.

Finally, to plan behavior to reduce diabetes risk, participants are guided toward developing a personalized SMART (Specific, Measurable, Achievable, Realistic, and Timebound) strategy for attending the NDPP. Coaches help participants identify their anticipated barriers to attendance (eg, need for child care) and possible solutions that would enable participation (eg, finding other caregivers or bringing children to class on occasion if needed). Participants are also encouraged that more frequent attendance is associated with greater weight loss, but overall benefits can be achieved despite missing some sessions: attending ≥15 sessions is associated with achieving the ≥5% weight loss goal on average (ie, each session is associated with 0.31% weight loss [4]). Scaling questions are used to help individuals identify an appropriate initial goal. For example, although some participants may have limited confidence to make a commitment to attend the yearlong NDPP without having tried it before, they may report a 10 of 10 in confidence to attend at least the first NDPP session. Finally, participants complete an individualized action plan that includes their SMART goal and anticipated problem-solving strategies. Coaches also conduct brief calls after presessions are completed to follow up and address remaining questions or concerns.

### Data Collection

The RCT focuses on comparing NDPP outcomes between participants who receive pre-NDPP versus direct enrollment into the NDPP. The assessment schedule is shown in Table 1.

Demographic characteristics are extracted from EHR databases and verified as needed during the first study visit, including age, gender, race and ethnicity, preferred language, income (above or below 133% of federal poverty level), and education (highest level completed). Body weight is measured on a high-capacity medical-quality scale at study visits and NDPP sessions. The primary outcome is percent weight change from baseline to 12 months by ITT analysis (without regard to whether participants declined NDPP or had early dropout). We also calculate percent weight change from the first to last NDPP sessions attended (ie, last observation carried forward), per CDC guidelines [17]. CMS standards for NDPP reimbursement also emphasize achieving ≥5% weight loss at any point in the program [24], assessed as a dichotomous outcome. Attendance in the NDPP is measured as ≥1 session attended, total number of NDPP sessions attended (including make-up sessions), and duration of participation in the yearlong program. Rates of completing between-session support calls are also assessed as an additional indicator of engagement and treatment dose. Per the CDC’s NDPP curriculum, participants self-report weekly minutes of moderate to vigorous physical activity since the last session [3]. Baseline BMI is also be assessed at the initial study visit as kg/m^2^.

We are assessing potential mediators of perceived risk and self-efficacy, key constructs of the Health Belief Model [8], and readiness for weight loss as an indicator of motivation. Perceived risk for developing diabetes is assessed with the Risk Perception Survey for Developing Diabetes, a 43-item Likert scale measure with four subscale scores on Comparative Disease Risk, Environmental Risk, Personal Control, and Optimistic Bias [20]. Self-efficacy for weight control is measured with the Weight Efficacy Lifestyle Questionnaire–Short Form, an 8-item measure of confidence (on a 10-point scale) managing five situational factors related to weight management behavior: negative emotions, availability, social pressure, physical discomfort, and positive activities [21]. We use validated Spanish-language versions of both measures [25,26]. Weight loss readiness is assessed with the Stages of Change in Overweight and Obese People (S-Weight), a 5-item survey developed concurrently in English and Spanish by expert consensus [22,23]. Mediators are measured during an initial assessment at the time of enrollment and 1 to 2 weeks before the first NDPP session (ie, immediately after presessions are completed for the pre-NDPP arm, and shortly before NDPP classes begin for the usual care NDPP arm). This is intended to determine whether pre-NDPP results in increased perceived risk, self-efficacy, and readiness compared with the usual care NDPP, and whether changes in these variables mediate outcomes.

We are also evaluating implementation factors regarding pre-NDPP using the Reach, Effectiveness, Adoption, Implementation, and Maintenance (RE-AIM) planning and evaluation framework for implementation research [27]. RE-AIM constructs will be assessed through a combination of recruitment data, intervention outcomes, staff logs, observations, and interviews with Lifestyle Coaches, clinic personnel, and patients, as well as cost records (Table 1).

Observations and interviews with Lifestyle Coaches and clinic personnel will focus on how pre-NDPP implementation works in practice and where gaps in care processes may be. Interviews will seek to understand the context of intervention delivery and mindsets and belief systems that drive thoughts and actions regarding pre-NDPP. External influences such as financial demands and staff turnover will also be explored as potential challenges. Both Lifestyle Coaches and 3 personnel per each of 8 clinics (focusing on high- and low-referring providers and clinic directors) will be interviewed. The qualitative research assistant will shadow presessions using the observation template and field notes to determine fidelity to core features of the pre-NDPP protocol. Patient interviews will focus on discerning similarities and differences in perspectives about the pre-NDPP and NDPP across 6 groups: those (1) randomized to pre-NDPP and (2) randomized to usual care, and within these groups, those (1) who initially decline to enroll in the NDPP, (2) who enroll but complete <6 months of the NDPP, and (3) who complete ≥6 months. We will begin with 5 interviews per group and continue until the thematic saturation is achieved (ie, not eliciting new information). Key interview questions include the extent to which pre-NDPP sessions increase motivation, relieve uncertainty about participating in the NDPP, address practical barriers to engagement, support autonomy, and other emergent factors that may influence participation. Patients and clinic personnel will be provided gift-card incentives. Interviews will be recorded with consent and transcribed for analysis.

We will measure pre-NDPP costs using principles of time-driven, activity-based costing [28,29], accounting for Lifestyle Coach and supervisor time (including salaries and benefits), supplies and other direct costs, and indirect costs (eg, facilities and general administrative expenses). To focus on the cost of delivering the pre-NDPP, we will exclude costs associated with standard NDPP delivery and research-related costs (eg, data collection solely for research purposes). To facilitate accurate estimates of personnel costs, coaches and their supervisor will track the time spent on pre-NDPP activities (eg, training, outreach, preparing for, and conducting pre-NDPP) and report total hours for each pre-NDPP activity quarterly. Supplies and other direct costs will also be reported quarterly.

**Table 1:** Measures for randomized controlled trial of Pre-National Diabetes Prevention Program

Measure		Description	Method of collection	Timeframe
**Characteristics**				
	Demographics	Age, gender, race and ethnicity, primary language, income, education	Collected from EHR^a^ and during first study visit	BL^b^
	BMI	Baseline BMI (kg/m^2^)	Collected during first study visit	BL^b^
**Main outcomes**				
	Initial NDPP^c^ attendance	≥1 NDPP session attended	Collected during NDPP delivery	Ongoing collection
	Number of sessions attended	1-25 NDPP sessions attended	Collected during NDPP delivery	Ongoing collection
	Duration in NDPP	1-365 days of NDPP participation	Collected during NDPP delivery	Ongoing collection
	Number of between-session calls completed	1-25 between-session support calls completed	Collected during NDPP delivery	Ongoing collection
	Physical activity	Average self-reported weekly minutes at each NDPP session	Collected during NDPP delivery	Ongoing collection
	Percent weight change	Based on (1) BL to 12 months (primary outcome), and (2) first to last NDPP sessions attended	Collected during NDPP delivery; note weight is also measured at an initial study assessment and at a final 12-month study visit (we are setting up a time for all randomized individuals to have their weight measured at these study visits, for which they will receive gift cards)	Ongoing collection
	≥5% weight loss	Achieved at any point in the NDPP	Collected during NDPP delivery	Ongoing collection
**Mediators**				
	Risk perception	Risk Perception Survey for Developing Diabetes [20]	Administered by Lifestyle Coaches during first study visit and repeated prior to start of NDPP classes to assess pre-post change	BL and 1-5 days prior to start of NDPP classes
	Self-efficacy	Weight Efficacy Lifestyle Questionnaire [21]	Administered by Lifestyle Coaches during first study visit and repeated prior to start of NDPP classes to assess pre-post change	BL and 1-5 days prior to start of NDPP classes
	Readiness	Stages of Change in Overweight and Obese People [22,23]	Administered by Lifestyle Coaches during first study visit and repeated prior to start of NDPP classes to assess pre-post change	BL and 1-5 days prior to start of NDPP classes
**Implementation factors using Reach, Effectiveness, Adoption, Implementation, and Maintenance**				
	Reach—Absolute number, proportion, and representativeness of individuals who participate	Number and characteristics of patients referred, outreached, and expressed interest, consented, completed pre-NDPP (intervention group), and attended NDPP (both groups); reasons for not enrolling or dropout	Demographics and referral data from EHR; enrollment and participation data collected by coaches; reasons for participating or declining collected by QRA^d^ interviews with select participants	Ongoing collection
	Effectiveness—Intervention impact on key outcomes	Based on main outcomes listed above	Based on main outcomes listed above	Based on main outcomes listed above
	Adoption—Absolute number, proportion, and representativeness of settings and agents willing to initiate intervention	Number and characteristics of participating Denver Health clinics; NDPP referrals; Lifestyle Coach participation Factors influencing adoption	Study documentation; EHR data QRA interviews with Lifestyle Coaches and select clinic personnel	Monthly abstraction analysis BL; 6 months after start of pre-NDPP

	Implementation—Fidelity to intervention protocol, including consistency of delivery (eg, bias) and time and cost of intervention	Completion of pre-NDPP and NDPP protocol components Acceptability of pre-NDPP components, processes, and tools; any adaptations made by coaches Process, barriers, facilitators to implementing pre-NDPP Pre-NDPP cost	Coach documentation; presession shadowing by QRA; fidelity checks Survey by QRA to Lifestyle Coaches, select clinic personnel and select patients QRA interviews with select participants, select clinic personnel and Lifestyle Coaches Lifestyle Coach time and resources survey	Ongoing collection 6 months after start of pre-NDPP 12 months after start of pre-NDPP Quarterly after each presession
	Maintenance (potential)—Extent to which intervention becomes routine practice and long-term participant benefits	Plans and intent to continue, or modify and adapt, pre-NDPP after study; ROI^e^ as an indicator of potential sustainability; 12-month weight loss outcomes	QRA interviews with Lifestyle Coaches and select clinic staff; document review and abstraction of NDPP payment schedules (eg, Medicare); above outcome data	Study completion

^a^EHR: electronic health record.

^b^BL: baseline.

^c^NDPP: National Diabetes Prevention Program.

^d^QRA: qualitative research assistant.

^e^ROI: return on investment.

### Analysis Plan

#### General Quantitative Approaches

Differences in characteristics between study arms will be assessed using chi-square and *t* tests to examine potential sampling bias. Percent weight change is the primary outcome, which has a well-documented association with T2D incidence [2,30]. ITT analyses will include all randomized participants regardless of NDPP participation, including those lost to follow-up. Weight loss data for women who become pregnant during the study will be excluded from analyses. Patient-level covariates will be screened in bivariate analyses and included in multivariate analysis if related to the outcome at *P*<.20, differ between treatment arms, or associated with dropout. Covariates and potential moderators will include age, gender, race and ethnicity, primary language, comorbidities, and other demographic and clinical variables. Although primary analyses examine a single outcome per patient (eg, percent weight loss), for longitudinal analyses (eg, perceived risk), we will determine whether missingness patterns are ignorable or nonignorable [31-34]. If so, we will employ likelihood-based methods that use all available data, adjusting for covariates associated with missingness. If missingness is nonignorable, we will use pattern mixture models [35]. If normality assumptions are not met, we will use transformations to normalize distributions, ordinal or Poisson regression where appropriate, and/or the appropriate link function and distribution (eg, logit link and gamma distribution). We will use general (generalized) linear mixed models to incorporate data structures that may be both hierarchical (patients within groups) and longitudinal (repeated observations over time) [36,37]. Hypothesis tests will be two-sided with α=.05 or *P* values reported. Goodness of fit statistics and model fitting diagnostics will be used to assess for influential points, outliers, overdispersion, and heteroscedasticity and to evaluate alternative model specifications [37]. Analysts will be unblinded to condition but will not conduct preliminary analyses to minimize potential of biasing other project staff. Further, while Lifestyle Coaches record individual participant weights and minutes of physical activity at each NDPP session (per standard CDC guidelines for NDPP delivery), they will not calculate aggregate outcomes at the cohort level. SAS version 9.4 (SAS Institute Inc) will be used for analyses.

#### Aim 1: To Evaluate Clinical Effectiveness of the Pre-National Diabetes Prevention Program Intervention

##### Hypothesis 1.1

Pre-NDPP participants will experience greater weight loss than those directly enrolled into NDPP. The primary outcome for this analysis will be percent weight change among all randomized participants. As study participation includes groups of individuals in the same study arm, the data structure will be hierarchical, with patients nested within groups. Statistical models are shown in hierarchical notation below. Likelihood of achieving ≥5% weight loss in the NDPP will also be evaluated (using generalized linear mixed models with logit link), as well as percent weight change from first to last NDPP sessions attended.

##### Hypothesis 1.2

Pre-NDPP participants will have greater engagement in the NDPP than those who are directly enrolled into the program. The outcome variables, number of sessions attended and days of participation, will be analyzed using similar approaches. If the distribution of outcomes is nonnormal, we will use generalized linear mixed models with the appropriate distribution and link function, as described earlier. We will also examine the dichotomous outcome of ≥1 NDPP session attended using multilevel logistic regression (generalized linear mixed model with logit link and random effect for group).

#### Aim 2: To Examine Mediators and Moderators of Pre-National Diabetes Prevention Program Outcomes

##### Hypothesis 2.1

The Pre-NDPP intervention will increase perceived risk for developing diabetes and self-efficacy and readiness for weight management. Outcomes for these analyses will be patients’ perceived risk, self-efficacy, and readiness scores over time. We will use longitudinal models to determine if trajectories differ for patients in control versus intervention groups.

##### Hypothesis 2.2

Perceived risk, self-efficacy, and readiness will mediate relationships between pre-NDPP treatment and outcomes. Outcomes will be weight loss and session attendance, using similar approaches as described earlier for hypotheses 1.1 and 1.2. We will include baseline perceived risk, self-efficacy, and readiness as covariates and change in these constructs as primary independent variables to determine if the intervention effect is partially or fully explained by these hypothesized mediators [38].

##### Hypothesis 2.3

Pre-NDPP effects will differ for participants with the moderator condition (eg, Hispanic and low-income) compared with those without the moderator (non-Hispanic and higher income). The effects of moderator analyses involve the inclusion of an intervention × moderator fixed effect for models that are not longitudinal (eg, percent weight loss and number of sessions attended). For longitudinal models (eg, self-efficacy over time), models will include a main effect for time, arm, moderator variable, time × arm, time × moderator, arm × moderator, and time × arm × moderator interaction term. The 3-way interaction term tests for differential intervention effectiveness in subgroups identified by the moderator variable.

### Sample Size and Power

Pre-NDPP pilot data indicate a 0.36 effect size for percent weight change in the NDPP with an intraclass correlation coefficient (ICC) of 1.44%. To be conservative for our primary outcome of percent weight loss among all randomized participants regardless of NDPP participation, we estimate minimum effect sizes detectable for various sample sizes and ICCs (Table 2), with effect sizes of approximately 0.28 to 0.35 for analyses of the primary outcome with a type 1 error rate of .05. Consequently, we expect that 500 randomized participants will provide adequate power while accounting for 20% potential attrition. Note that mediation and moderation analyses are considered exploratory, as estimated power is unknown.

**Table 2:** Estimated power to detect treatment differences in percent weight loss.

Groups per arm	Patients per group	Intraclass correlation coefficient (%)	Effective sample size	Detectable difference (effect size)	Power (%)
10	18 (180 per arm)	1	154	0.32	80
10	18 (180 per arm)	2	134	0.35	82
10	20 (200 per arm)	1	168	0.31	81
10	20 (200 per arm)	2	145	0.33	80
12	20 (240 per arm)	1	201	0.28	80
12	20 (240 per arm)	2	174	0.31	82

### Secondary Analyses

Secondary analyses will be used to inform future applications of pre-NDPP. We will examine whether pre-NDPP participants only benefit if they attend NDPP thereafter (vs no benefit for those not attending NDPP after presessions), which would suggest potential utility as a screening process to identify individuals likely to engage in and benefit more from the NDPP. Additional secondary analyses will be conducted to determine whether patient characteristics that are associated with poor outcomes (ie, lack of retention and little or no weight loss) differ between control and intervention participants. If so, this information can be used to better target the intervention to individuals who could benefit from the presession.

#### Aim 3: To Evaluate the Implementation Factors of Pre-National Diabetes Prevention Program

Qualitative analyses will evaluate pre-NDPP implementation from a Lifestyle Coach, clinic provider and leadership, and patient perspective. Interviews and observation data will be cleaned and entered into the qualitative software program ATLAS.ti (version 8; Scientific Software Development GmbH) for analysis. Analyses will begin as a small group process for data triangulation to occur and use a grounded hermeneutic editing approach [39]. Qualitative researchers will read 5 to 10 interviews and together determine key themes and their definitions and labels (*codes*). Codes will be vetted with the larger study team and stakeholder representatives. After establishing initial codes, analysts will code the data (first together, then independently) as outlined by Addison [39] and will compare and reconcile coding until a high degree (≥80%) of conceptual interrater reliability is achieved. Specifically, data from interviews with Lifestyle Coaches and clinic personnel will examine themes related to adoption, feasibility, and acceptability of pre-NDPP. This analysis will determine key underlying characteristics, such as belief systems or mindsets, and/or practical reasons that make pre-NDPP effective or not, and to what extent. We expect that data from patient interviews will more thoroughly explain engagement in the NDPP. We will examine emergent codes across study groups by comparing group-level quotations to determine differential experiences. Finally, perceived reasons for participation (or nonparticipation) will be examined alongside actual engagement data to corroborate and explain quantitative results. In ongoing meetings with the larger study team, we will further consider existing literature and associated experiences for corroboration and seek out additional data as needed to confirm or refute results. After initial analysis has identified data to support one theme or interpretation, effort will be devoted to finding negative or disconfirming evidence. Clinic personnel and Lifestyle Coaches will be selected for member checking and revision of thematic groupings before final coding. The final phase consists of preparing interpretive summaries detailing the findings of prior phases. All phases of data processing and analysis will be cross-checked to ensure consistency in application of coding and classification procedures. Observation data will be analyzed similarly.

#### Pre-National Diabetes Prevention Program Cost and Return on Investment

We will calculate pre-NDPP cost as the average expense of each presession delivery based on personnel time, supplies and other direct costs, and indirect costs. We will then determine the projected return on investment (ROI) of pre-NDPP from both provider and payer perspectives. For NDPP providers, ROI will be calculated as the additional payment expected from payers as a result of potentially improved retention and weight loss of pre-NDPP participants minus the average presession cost and divided by presession cost. For common reference, payments will be based on the Medicare reimbursement schedule for achievement of NDPP attendance and weight loss milestones [24]. We will compare the average expected reimbursement for participants in both study arms to measure additional payments that may be attributed to pre-NDPP. We will also conduct a sensitivity analysis by calculating the projected ROI for varying numbers of pre-NDPP participants with varying demographic characteristics (eg, race and ethnicity and income) and with other available NDPP payment schedules (eg, Maryland Medicaid) [40]. Sensitivity analysis results will inform pre-NDPP sustainability by identifying the number of participants needed per presession to achieve a positive ROI, the extent to which moderators identified in hypothesis 2.3 affect ROI, and the extent to which different payment models affect ROI. From the perspective of NDPP payers, ROI will account for the expected reduction in direct health care expenditures as a result of covering pre-NDPP through an additional payment to NDPP providers, as calculated over a 3-year horizon. ROI will be the reduction in projected expenditures minus the average presession cost and divided by presession cost. Estimates of change in direct health care expenditures will be based on the impact of pre-NDPP on weight loss from hypothesis 1.1, the known relationship between weight loss and T2D incidence [30], and the difference in expenditures for individuals with prediabetes or T2D over a 3-year horizon [41]. This timeline requires discounting of expected reductions in years 2 and 3 expenditures for which we will apply a standard 3% discount rate. We will also conduct a sensitivity analysis by varying the number of pre-NDPP participants, their characteristics, and the discount rate. To be conservative, cost and ROI are based on all pre-NDPP participants, regardless of NDPP attendance.

## Results

Recruitment is underway as of July 2019. Initial participants will begin the intervention in October 2019. Data analysis and results reporting is expected to be completed in 2024.

## Discussion

### NDPP Outcomes

NDPP outcomes are suboptimal, especially for disadvantaged populations, which involves the risk of further widening of health disparities if not addressed. Pilot data show feasibility and promising results of pre-NDPP among diverse, underserved patients with elevated diabetes risks, but are limited by small sample size of the intervention group and no concurrently randomized control group. To address the research gap, we are now conducting an RCT to compare NDPP attendance and weight loss among diverse, predominately underserved participants who receive pre-NDPP versus direct enrollment into NDPP (usual care).

The primary hypothesis is that presessions improve NDPP engagement and weight loss, which will be confirmed if those randomized to pre-NDPP have better outcomes than those receiving usual care NDPP. A secondary analysis will examine whether only pre-NDPP participants who go on to NDPP benefit (vs no benefit for those declining NDPP), which would suggest that presessions screen for individuals likely to participate adequately, and thus benefit from, the NDPP. Screening via presessions may yet be an efficient population health strategy to (1) increase risk awareness for the estimated one-third of US adults with prediabetes [1], (2) offer informed decision-making, and (3) maximize performance-based reimbursement for suppliers, which supports access [42]. In either case, a brief group model may be optimal as (1) individual presessions appear cost-prohibitive, while longer sessions may also be more taxing on vulnerable populations; (2) uptake by NDPP suppliers likely depends on establishing efficacy in a low-cost, high-reach model; and (3) a key goal is supporting engagement in the yearlong NDPP for continued intervention, and thus increasing familiarity with its hour-long, group class format may be important.

If effective, greater uptake is expected if NDPP providers and payers can understand how pre-NDPP achieves an effect and whether their populations are likely to benefit, which will be addressed via mediation and moderation analyses. To prepare for future dissemination, we will also evaluate implementation factors, including cost of adding presessions to NDPP delivery and estimated RO. If effective, this approach may reduce disparities in NDPP effectiveness. It can also be disseminated to all NDPP providers, including more than 1700 suppliers [43], and may be supported by current NDPP payers such as Medicare, commercial insurers, and employer groups [24,44,45]. Thus, pre-NDPP has potential for high impact on the burden of T2D and related health disparities across the country.

#### Limitations

This study is powered on percent weight loss; more limited power is expected to evaluate pre-NDPP effectiveness among demographic subgroups and mediators and moderators. Although we do not anticipate difficulty meeting recruitment goals from provider referrals, we can identify additional eligible participants as needed from DH’s EHR. Recruitment is limited to a single health care system, yet in a variety of different clinics and following CDC standards for NDPP delivery. Our study requires initial contact by phone to proceed with enrollment, thus we may be systematically missing especially under-resourced individuals who lack sufficient connectivity. Further, participants are initially assigned to pre-NDPP or usual care NDPP, yet some pre-NDPP participants may not attend the presession prior to beginning NDPP classes, which may result in a lower effect size than observed in our pilot study of outcomes following presession completion.

Lifestyle Coaches are necessarily unblinded to condition, thus introducing potential to bias their delivery of pre-NDPP and usual care NDPP interventions. While Lifestyle Coaches follow a standardized intervention manual for pre-NDPP, exact delivery of components like MI techniques may vary among presessions, as coaches must be responsive to the unique presentation of each group of pre-NDPP participants. Similarly, variability may occur for delivery of the NDPP curriculum across cohorts.

Economic analysis limitations include reliance on the literature-derived estimates of projected cost savings and the relationship between weight loss and T2D incidence. It is possible that there will be limited or no effect of pre-NDPP in an RCT, but pilot results are strong, and any clinically meaningful benefit may be worthwhile given pre-NDPP is expected to be a relatively low-resource intervention. Financial incentives may in fact lead to better outcomes than obtained in previous observational study but are only offered for study-related assessments and appropriately sized.

In summary, this RCT of pre-NDPP may lead to future dissemination of a scalable, evidence-based strategy to improve success of the NDPP, reduce disparities in NDPP effectiveness, and help prevent T2D across the country.

## Appendix

Multimedia Appendix 1 Peer-reviewer report from NIH.

## Abbreviations

CDC: Centers for Disease Control and Prevention
DH: Denver Health
EHR: electronic health record
GDM: gestational diabetes mellitus
ICC: intraclass correlation coefficient
ITT: intent-to-treat
MI: motivational interviewing
NDPP: National Diabetes Prevention Program
NHB: non-Hispanic black
NHW: non-Hispanic white
pre-NDPP: presession prior to the NDPP
RCT: randomized controlled trial
RE-AIM: Reach, Effectiveness, Adoption, Implementation, and Maintenance
ROI: return on investment
SMART: Specific, Measurable, Achievable, Realistic, and Timebound
T2D: type 2 diabetes
