# Supplementary material for: Enhanced Enrollment in the National Diabetes Prevention Program to Increase Engagement and Weight Loss for the Underserved: Protocol for a Randomized Controlled Trial
Source: JMIR Res Protoc. 2020 Jun 1;9(6):e15499. doi: 10.2196/15499 (PMC7296404; doi:10.2196/15499)
Supplement: Multimedia Appendix 1 [file resprot_v9i6e15499_app1.pdf]

SUMMARY STATEMENT
(Privileged Communication)

PROGRAM CONTACT:
Pamela Thornton
301.480.6476
thorntonpl@niddk.nih.gov

Release Date: **10/25/2018**
Revised Date:

Application Number: **1 R01 DK119478-01A1**

Principal Investigator

RITCHIE, NATALIE DAWN

Applicant Organization: DENVER HEALTH AND HOSPITAL AUTHORITY

Review Group: **DIRH**
Dissemination and Implementation Research in Health Study Section

Meeting Date: **10/10/2018**
Council: **JAN 2019**
Requested Start: **04/01/2019**
RFA/PA: **PA18-330**
PCC: **DPT HET2**

Project Title: **Enhanced enrollment in the National Diabetes Prevention Program for the underserved: a randomized control trial**
SRG Action: **Impact Score:20 Percentile:9**
Next Steps: **Visit https://grants.nih.gov/grants/next_steps.htm**
Human Subjects: 48-At time of award, restrictions will apply
Animal Subjects: 10-No live vertebrate animals involved for competing appl.
Gender: 1A-Both genders, scientifically acceptable
Minority: 1A-Minorities and non-minorities, scientifically acceptable
Children: 3A-No children included, scientifically acceptable

Project Year	Direct Costs Requested	Estimated Total Cost
1	452,166	664,979
2	475,244	698,919
3	491,177	722,351
4	476,231	700,370
5	411,032	604,485
TOTAL	2,305,850	3,391,104

ADMINISTRATIVE BUDGET NOTE: The budget shown is the requested budget and has not been adjusted to reflect any recommendations made by reviewers. If an award is planned, the costs will be calculated by Institute grants management staff based on the recommendations outlined below in the COMMITTEE BUDGET RECOMMENDATIONS section.
EARLY STAGE INVESTIGATOR
NEW INVESTIGATOR

1R01DK119478-01A1 Ritchie, Natalie

EARLY STAGE INVESTIGATOR
NEW INVESTIGATOR
PROTECTION OF HUMAN SUBJECTS UNACCEPTABLE

RESUME AND SUMMARY OF DISCUSSION: This application proposes to conduct a randomized controlled trial to assess the effectiveness and implementation factors, as well as examine mediators and moderators, of the Pre-National Diabetes Prevention Program (Pre-NDPP), compared to direct enrollment into the NDPP, on NDPP engagement and weight loss among racial/ethnic minority and low-income populations with elevated diabetes risks. The panel agreed that, if successful, the application would improve the percent weight loss, as well as reduce type 2 diabetes and related health disparities, among underserved populations enrolled in the NDPP. During the discussion, the panel agreed that the application was highly significant, and the scientific premise was strong as evidenced by the literature and preliminary data demonstrating efficacy of the Pre-NDPP intervention, with the use of motivational interviewing (MI), in increasing participant retention in the NDPP. This resubmission is highly responsive to the prior critiques with the addition of a literature supporting the group MI format, addition of the Weight Loss Readiness assessment and more details regarding the cost-effectiveness and projected return on investment. This is an outstanding multidisciplinary team with stellar backgrounds, a collaborative history, strong experience and a supportive environment to achieve the study's aims. The panel viewed the idea of using a pre-intervention counseling session to increase retention to the NDPP, as somewhat innovative. Reviewers identified several strengths, including: having a comprehensive theoretical conceptual framework, a detailed cost-effectiveness analysis plan and using a mixed methods approach, strengthening the scientific rigor. The panel also noted the limited consideration of sex as a biological variable, broad inclusion criteria and a lack of dietary self-monitoring to support weight loss, as weaknesses. There is also a protection of human subjects' concern. Following the discussion, reviewers viewed these weaknesses as minor, easily addressable and did not detract from the application's high merit. Overall, the panel expressed high enthusiasm for the application and agreed that the findings are expected to have a high impact on increasing the retention of underserved populations in the National Diabetes Prevention Program and reducing the prevalence of type 2 diabetes and related health disparities.

DESCRIPTION (provided by applicant): Type 2 diabetes affects 9.4% of US adults with higher rates among racial/ethnic minorities and individuals of low socioeconomic status. The National Diabetes Prevention Program (NDPP) is an evidence-based and widely disseminated behavioral intervention to reduce diabetes incidence through modest weight loss. However, retention in the yearlong NDPP is problematic and leads to suboptimal weight loss, especially among Hispanic, non-Hispanic black, and low-income non-Hispanic white participants. Strategies to improve NDPP engagement and weight loss are needed urgently, especially for these subgroups. Pilot results of the Pre- NDPP, a novel enhancement to enrollment in the NDPP based on the Health Belief Model, were highly successful in a non-randomized cohort study among 1,140 racially diverse, predominately low-income participants. Outcomes of 75 Pre-NDPP participants who enrolled in the NDPP were compared to 1,065 prior participants using ANCOVA and multivariable logistic regression. Pre-session participants stayed in the NDPP 99.8 days longer ($p<.001$) and attended 14.3% more sessions ($p<.001$) on average than those without a pre- session. Pre-session participants lost 2.0% more weight ($p<.001$) and were 3.5 times more likely to achieve the 5% weight loss target ($p<.001$). Sensitivity analyses were consistent. Findings suggest pre-sessions may be a promising and pragmatic strategy to improve NDPP effectiveness and mitigate disparities in program outcomes, but a randomized controlled trial (RCT) is needed to determine whether Pre-NDPP reliably improves NDPP outcomes. The purpose of this study is to 1) conduct an RCT comparing NDPP attendance and weight loss outcomes between participants who receive Pre-NDPP vs. direct enrollment into the NDPP (usual care), 2) examine potential effect mediators (perceived risk for developing diabetes and self-efficacy and readiness for weight control) and moderators (race/ethnicity and income level), and 3) evaluate implementation factors, including

cost and projected return on investment. The long-term goal is to disseminate a scalable, evidence-based strategy to improve success of the NDPP and reduce disparities in NDPP effectiveness. If found to be effective, Pre-NDPP can be disseminated to all NDPP providers, including more than 1,700 NDPP sites, and may be supported by current NDPP payers such as Medicare, commercial insurers, and employer groups. Thus, this approach has a high potential to impact the burden of type 2 diabetes and related health disparities across the country.

PUBLIC HEALTH RELEVANCE: The National Diabetes Prevention Program (NDPP) is a widely available, evidence-based intervention that promotes weight loss to prevent type 2 diabetes; however, participant attendance is problematic and leads to suboptimal weight loss, especially among Hispanic, non-Hispanic black, and low-income non-Hispanic white participants. An innovative pre-session enhancement to the NDPP (Pre-NDPP) showed successful results upon initial application in a diverse and predominately low-income population, with doubled attendance and weight loss outcomes as compared to previous NDPP participants who did not receive a pre-session. If Pre- NDPP is shown to be successful upon more rigorous study, it can be widely adopted by NDPP providers across the country to help reduce diabetes prevalence and related health disparities.

CRITIQUE 1

Significance: 1
Investigator(s): 1
Innovation: 3
Approach: 3
Environment: 1

Overall Impact: The purpose of the proposed study is to increase the retention of racial/ethnic minority and lower SES NDPP participants while assessing factors that may impact future Pre-NDPP dissemination and implementation. The investigators will use a randomized design to evaluate the effects of a pre-NDPP intervention which provides information on diabetes risk and utilizes motivational interviewing prior to enrollment compared to usual care (no motivational interviewing) and to examine the effect moderators/mediators, a cost effectiveness analysis is also included. If successful, the intervention to increase the NDPP effectiveness among these groups addresses a significant problem given their known disparities in obesity and lower weight loss. The application is a resubmission; the investigators have been highly responsive to the previous reviewers' concerns. The proposed research has a strong scientific premise as evidenced by the NDPP's demonstrated scalability and literature indicating that most NDPP sites are unable to meet the program goals of 5% weight loss, retention and outcomes are lower among racial/ethnic minorities and lower SES. Additionally, the literature supporting that session attendance predicts weight loss and that motivational interviewing has been shown to promote motivation for health behavior change further indicate that the proposed intervention is based on sound scientific premise. The environment is excellent as Denver Health is a leader and major stakeholder of successful NDPP interventions. The early-stage PI is productive and has the training and experience to lead the project and has composed an experienced team of co-investigators. Sex as a biological variable is addressed in the analysis plan, there is no discussion of reporting data disaggregated by gender. The proposed mixed methods approach is not technically innovative, yet the approach and evaluation plans are appropriate and well thought out. A few score driving weaknesses that can be easily addressed are noted in the approach, otherwise the application is strong. Successful completion of the aims has the potential for high impact on the DPP field as cost effective methods to retain diverse participants and enhance an evidence-based, scaled intervention will impact nation-wide diabetes prevention efforts.

1. Significance:

Strengths

- The proposal clinical trial is necessary to advance the DPP field by increasing the attendance and intervention effectiveness of disparate populations. It has the potential to advance clinical practice and influence policy.
- The NDPP has shown wide spread scalability, increasing the effectiveness of dissemination by increasing adherence and attendance among individuals most at risk of type 2 DM is warranted. If the aims of the proposed project are met the effectiveness of the NDPP could be improved which has the potential for high public health impact.
- The proposal is based on sound scientific premise.
- Denver Health is a leader of the NDPP therefore they are major stakeholders in the successful implementation of the project.
- The NDPP and motivational interviewing have evidence of effectiveness, thus the proposed project is based on sound scientific premise.
- Including the cost effectiveness fills a significant gap in the DPP literature.

Weaknesses

- None noted

2. Investigator(s):

Strengths

- The team is well suited for the proposed work
- The early stage investigator has the training and experience to complete the work, moreover she has composed a team of more senior scientist and mentors with extensive experience to assist with meeting all project milestones.
- The PI is bilingual thus able to adapt the curriculum for Hispanic-speaking participants.
- The team is productive and has a history of previous collaboration
- Team includes D&I experts.
- Team includes cost analysis expert.

Weaknesses

- None noted

3. Innovation:

Strengths

- The pragmatic approach is not particularly innovative; however, the intervention may provide information that will advance the field of DPP in a cost-effective manner.
- Examining cost and ROI from NDPP payer and provider perspectives
- Adding a pre-session prior to enrollment into the NDPP is a shift to current practice.

Weaknesses

- None noted

4. Approach:

Strengths

- Detailed description and pilot testing of the theory-driven Pre-NDPP with stakeholder involvement that included previous NDPP participants and coaches
- The study design is generally well thought out and rigorous. Although the pre-sessions are labor intensive, their potential to cost-effectively increase participant retention and weight outcomes will have high impact on the DPP field.
- The investigator's previous work makes the proposed project a logical next step
- Theoretical underpinning with the Health Belief Model is well-integrated in the intervention and evaluation plan.
- The results of the pilot study are compelling for Aim 1.
- Randomizing the lifestyle coaches to deliver NDPP and Pre-NDPP with accompanying fidelity
- Utilizing block sizes and stratification by demographics increases the likelihood of enrolling the target population
- Comprehensive qualitative approach will elicit rich data for future implementation.
- Randomly assigning the lifestyle coach controls potential confounders.
- Well defined cost effectiveness measures accounts for “real world” application of the pre-NDPP activities; addition of literature-supported justification for low-cost group-based MI vs individual
- Detailed Re-AIM evaluation plan using mixed methods is thorough.
- Attempting to reach lost to follow-up and determining the reasons for declining participation is responsive to previous reviewers and strengthens Aim 3.
- The addition of Weight Loss Readiness assessment is responsive to previous reviewers concerns regarding internal validity.

Weaknesses

- Major: The use of MI is a major factor of the pre-session, therefore the overall training of the coaches, especially the MI training warrants more details, e.g. length, content, etc.
- Moderate. Sex as a biological data warrants more discussion.
- Minor: The logistics of using the pre-diabetes screening tool as an inclusion criteria warrants more detail

5. Environment:

Strengths

- The proposed clinical site is a national leader in the NDPP and have provided a letter of support, thus they are major stakeholders in the success of the proposed project.
- The University has the resources, facilities and equipment for the project.
- The clinical site serves a diverse population and a current referral process, therefore the probability of reaching the target population is high.
- Offering the sessions at neighborhood clinics reduces the travel burden.
- The availability of bilingual lay health educators improves the intervention delivery and ensures Spanish adaptation and culturally-appropriate delivery.

Weaknesses

- None noted

Study Timeline:

Strengths

- The activities are doable within the grant period.
- Details all aspects of project implementation, including start- up activities.
- The timeline uses the existing resource of the health center and electronic medical records.

Weaknesses

- None noted

Protections for Human Subjects:

Acceptable Risks and/or Adequate Protections

- The study is minimal risk. All participants will provide consent and participant privacy is protected.

Data and Safety Monitoring Plan (Applicable for Clinical Trials Only):

Acceptable

- Data safety monitoring plan includes an independent safety monitor as well as plans for a Data and Safety Board if indicated during the implementation of the project.

Inclusion of Women, Minorities and Children:

- Sex/Gender: Distribution justified scientifically
- Race/Ethnicity: Distribution justified scientifically
- For NIH-Defined Phase III trials, Plans for valid design and analysis: Not applicable
- Inclusion/Exclusion of Children under 18: Excluding ages <18; justified scientifically
- The focus on adults with pre-diabetes is justification for excluding children.

Vertebrate Animals:

Not Applicable (No Vertebrate Animals)

Biohazards:

Not Applicable (No Biohazards)

Resubmission:

- The investigators were highly responsive to the reviewer concerns by providing more literature support of the merits of the group MI format; detailing the qualitative approach; clarifying the Denver Health pilot data; adding ROI from payer and provider perspective and revising the outcomes to measure call completion and participant readiness.

Resource Sharing Plans:

Not Applicable (No Relevant Resources)

Budget and Period of Support:

Recommend as Requested

CRITIQUE 2

Significance: 1
Investigator(s): 1
Innovation: 3
Approach: 3
Environment: 1

Overall Impact: The proposed study intends to determine the impact of 1 pre-intervention counseling session on participant engagement, retention, and weight loss compared to the standard NDPP intervention. The premise of the study is sound based on findings from the preliminary study. The necessary resources are available for study completion. Few outcomes will be measured and no assessment of the impact of the two approaches on glucose levels (the ultimate indicator of diabetes) will occur. However, the modification to the standard approach is modest in terms of time commitment and expertise needed for implementation. This modest adjustment, if effective, has the potential to be scalable and cost effective across a variety of providers. Successful completion of the study aims has the potential for high impact on disseminating diabetes prevention interventions.

1. Significance:

Strengths

- The application aims to improve rates of retention in NDDP programs which, if effective, could be disseminated to other NDDP providers across the U.S.
- The intervention will be delivered to a diverse population of adults at-risk for type 2 diabetes.
- Findings could influence reimbursement for NDDP participation by Medicare, commercial insurers, and/or employers.
- The adaptation proposed to the intervention is relatively low cost, minimally intensive, and could be disseminated by health coaches or lay educators.

Weaknesses

- None noted.

2. Investigator(s):

Strengths

- The research team has extensive experience in dissemination and implementation work in the field of diabetes prevention.
- The team has a history of prior collaborations and is well-rounded in terms of complimentary expertise.

Weaknesses

- None noted.

3. Innovation:

Strengths

- This modest modification to the NDPP intervention is pragmatic and potentially scalable in terms of diabetes prevention.

Weaknesses

- Addressing diabetes risk, readiness to change, and barriers to program attendance during the pre-session is not novel per se (minor).

4. Approach:

Strengths

- Preliminary data from a pilot study support the hypothesis that a pre-intervention counseling session will improve participant engagement and retention.
- Bilingual lay health educators are present and trained in the intervention.
- New NDPP sessions begin every 3-6 months in primary care clinics. Prior data suggests they can achieve the necessary sample size.
- Referrals to NDPP currently occur for high-risk patients who meet the eligibility criteria, indicating a viable avenue for recruitment.
- Cost effectiveness will be determined.

Weaknesses

- Only modest weight loss (mean of 3.5%) occurred among participants who completed the pre-session counseling prior to beginning the intervention in prior research. This level of weight loss is less than what is often achieved following the NDPP intervention (major).
- Inclusion criteria are extremely broad and do not adequately exclude individuals with potentially confounding or risk factors. For example, people who report a history of weight loss in the preceding 3-6 months are not excluded, which could reduce the amount of weight these individuals will lose during the study. Women who are pregnant or planning on becoming pregnant, those planning on bariatric surgery, and individuals with a history of cardiovascular disease, who may be at risk with greater levels of physical activity, also are not excluded (major).
- Participants complete a follow-up behavioral assessment by phone 1-5 days before the intervention begins. It’s not clear what assessments are conducted during this phone session (minor).
- The goal of the intervention is to achieve meaningful weight loss by achieving negative energy balance (e.g., greater physical activity and lower calorie intake). A low-fat diet is encouraged as part of the intervention, but participants will not be asked to self-monitor calorie and/or fat intake. While CDC may not require dietary self-monitoring, self-monitoring has been shown repeatedly to be one of the most effective strategies for promoting weight loss. It is difficult for the health coaches to determine how well participants are modifying their dietary intake without any monitoring during the intervention. Further, CDC material includes a food log for self-monitoring (major).
- The pre-session includes discussion of diabetes risks, an overview of type 2 diabetes, and guidance regarding the need for weight loss. The first session of the NDPP intervention includes this information as well. Thus, participants who are randomized to the standard intervention will receive some of the information delivered during the pre-session for the alternate treatment group. The duplication of information across both treatment groups minimizes true differences between the approaches (moderate).
- Potential mediators (risk perception, self-efficacy, and readiness to change) will only be collected at two timepoints (i.e., baseline and several days prior to initiation of NDPP). It seems reasonable to assess risk perception and self-efficacy at 12-month follow-up to determine whether the change between the 2nd timepoint and study end also impact weight change.

Adding a third assessment does not greatly increase participant burden given the small number of questionnaires proposed. Higher levels of self-efficacy have been shown in prior research to be associated with greater weight loss (moderate).

- No assessment of the impact of the interventions on the change in glucose will occur (minor).
- Sex as a biological variable is not specifically addressed (moderate).

5. Environment:

Strengths

- The necessary facilities and resources are available for project completion across an academic medical center and academic health sciences center.
- The Dissemination & Implementation program at UC Denver is a strength of the application.

Weaknesses

- None noted.

Study Timeline:

Strengths

- Adequate time is available for project completion with year 5 spent in data analyses and manuscript preparation.

Weaknesses

- None noted.

Protections for Human Subjects:

Acceptable Risks and/or Adequate Protections

Data and Safety Monitoring Plan (Applicable for Clinical Trials Only):

Unacceptable

- Adverse events should be assessed at each participant encounter and not simply expect participants to report illness or change in well-being.

Inclusion of Women, Minorities and Children:

- Sex/Gender: Distribution justified scientifically
- Race/Ethnicity: Distribution justified scientifically
- For NIH-Defined Phase III trials, Plans for valid design and analysis: Not applicable
- Inclusion/Exclusion of Children under 18: Excluding ages <18; justified scientifically
- No justification was provided for excluding individuals < 18 years old, but it is assumed that a separate intervention would be needed for adolescents.

Vertebrate Animals:

Not Applicable (No Vertebrate Animals)

Biohazards:

Not Applicable (No Biohazards)

Resubmission:

- The investigators adequately responded to critiques from the original submission.

Applications from Foreign Organizations:
Not Applicable (No Foreign Organizations)

Select Agents:
Not Applicable (No Select Agents)

Resource Sharing Plans:
Acceptable

Authentication of Key Biological and/or Chemical Resources:
Not Applicable (No Relevant Resources)

Budget and Period of Support:
Recommend as Requested

CRITIQUE 3

Significance: 1
Investigator(s): 1
Innovation: 3
Approach: 3
Environment: 1

Overall Impact: The purpose of the study is to conduct a pragmatic clinical trial to test whether the additional of a brief motivational intervention can improve retention in the National Diabetes Prevention Program, a widely disseminated, evidence-based intervention endorsed by the CDC. Retention in the NDPP is low in minority and low-income populations and the public health impact of the adapted intervention would be high if it improved retention in light of the potential ease of implementation of the modified NDPP. The study premise- that the modified intervention can increase retention and improve health outcomes- is supported by both compelling preliminary data and a large literature supporting the effects of brief motivational interventions to promote engagement in care and behavior change. The investigator team is strong and has experience both with NDPP program development and evaluation as well as behavioral health research overall. The environment is appropriate to the conduct of the study. Strengths of the approach include the use of an appropriate and rigorous design (effectiveness-implementation hybrid trial) that will allow for testing of the modified NDPP and identification of barriers to implementation of the new program if effective. The investigators were highly responsive to the prior reviews. A few minor weaknesses in the approach include the plan to have lifestyle coaches (interventionists) conduct study data collection, which could introduce bias and increase rates of missing data. The study is characterized by a number of significant strengths and a few minor, addressable weaknesses and the overall impact of the study on research in the area of behavioral interventions to prevent diabetes would be high.

1. Significance:

Strengths

- The need for this pragmatic clinical trial is supported by data showing high rates of drop-out from community-based NDPP programs, particularly among low-income and minority participants
- If successful, the study would have high public health impact by increasing NDPP retention among the highest-risk participants
- Modification of the NDPP by the addition of a single, group-delivered session of motivational interviewing (MI) would provide an easily scalable way to increase retention, if successful
- The premise of the study is supported by strong preliminary data from the applicant regarding the efficacy of the Pre-NDPP program when tested in a non-randomized content as well as the large body of literature showing that even single dose MI can result in moderate levels of behavior change

Weaknesses

- None noted.

2. Investigator(s):

Strengths

- The PI is an early stage investigator who has relevant experience and training to conduct the study, including the pilot study on which the current study is predicated and other studies on factors affecting NDPP implementation success
- The study co-investigators are senior behavioral health researchers with relevant experience in behavioral clinical trials.
- The study team includes both a biostatistician and a cost analyst.
- Several members of the team collaborated on the prior pilot study.

Weaknesses

- None noted.

3. Innovation:

Strengths

- Use of group-based MI as compared to individually delivered sessions is somewhat innovative.

Weaknesses

- The use of an effectiveness-implementation hybrid design is not particularly innovative although it is appropriate to addressing the study aims (Minor)

4. Approach:

Strengths

- The study design and methods are clearly articulated, increasing study rigor. The effectiveness-implementation design will allow critical barriers and facilitators to the future use of the Pre-NDPP program to be identified
- Plans to conduct qualitative interviews with a variety of stakeholders (participants, lifestyle coaches, clinic staff and administrators) will help to increase the likelihood of future dissemination of the adapted intervention

- The availability of a large pool of eligible participants at the recruitment site and history of enrolling 300+ participants per year in NDPP demonstrates that the planned sample can be recruited
- The applicants provide compelling preliminary data to support proceeding to a large sample RCT
- Treatment fidelity will be monitored through structured observations of the Pre-NDPP sessions
- A detailed analytic plan and power analyses were provided
- Risks to human subjects are minimized through appropriate protections and procedures

Weaknesses

- The lifestyle coaches will function as the outcomes assessors and will gather the study data. While this approach is sometimes necessary in pragmatic trials, a compelling case is not made in the application for why this would be required in the current study. Use of unblinded assessors who provide the intervention to participants could inject bias and reduces study rigor (Minor)
- While the applicants state that analyses will be intent to treat and that missing data will be imputed, the plan to use the interventionists as data collectors rather than independent research staff may increase the likelihood that there will be a significant amount of missing data. Imputation of large amount of missing data would reduce study rigor, especially as weigh loss is confounded with treatment attendance (Minor)

5. Environment:

Strengths

- Denver Health was an early adopter of the NDPP and provides an excellent clinical environment for the conduct of the study.
- Denver Health and UCSM will provide multiple relevant resources for the conduct of the clinical trial, including the Clinical and Translational Sciences Institute and support for early stage investigators through the DH Research Scholars Program

Weaknesses

- None noted.

Study Timeline:

Strengths

- The study start-up activities and clinical trial components can be completed within the proposed timeline and there is time in year 5 to extended treatment if recruitment goes more slowly than expected.

Weaknesses

- None noted.

Protections for Human Subjects:

Acceptable Risks and/or Adequate Protections

- The study is minimal risk, involving primarily standard-of care, behavioral interventions for weight loss in persons with overweight or obesity and has the potential for direct benefit to participants.

Data and Safety Monitoring Plan (Applicable for Clinical Trials Only):

Acceptable

- The PI will monitor adverse events given the intervention is largely standard of care and will report adverse events to the IRB and NIDDK in accordance with federal regulations

Inclusion of Women, Minorities and Children:

- Sex/Gender: Distribution justified scientifically
- Race/Ethnicity: Distribution justified scientifically
- For NIH-Defined Phase III trials, Plans for valid design and analysis: Not applicable
- Inclusion/Exclusion of Children under 18: Excluding ages <18; justified scientifically
- Women and minorities will comprise the majority of participants in the clinical trial. Children are excluded, as the DPP was developed and tested for adults

Vertebrate Animals:

Not Applicable (No Vertebrate Animals)

Biohazards:

Not Applicable (No Biohazards)

Resubmission:

- The resubmission addresses the major concerns raised by the prior reviewers including providing additional rationale for the use of single session, group delivered MI, details regarding MI training and monitoring of treatment fidelity and addition of measures of change in motivation as a mediator of intervention effects.

Resource Sharing Plans:

Not Applicable (No Relevant Resources)

Authentication of Key Biological and/or Chemical Resources:

Not Applicable (No Relevant Resources)

Budget and Period of Support:

Recommend as Requested

THE FOLLOWING SECTIONS WERE PREPARED BY THE SCIENTIFIC REVIEW OFFICER TO SUMMARIZE THE OUTCOME OF DISCUSSIONS OF THE REVIEW COMMITTEE, OR REVIEWERS' WRITTEN CRITIQUES, ON THE FOLLOWING ISSUES:

PROTECTION OF HUMAN SUBJECTS: UNACCEPTABLE

There were concerns regarding the protection of human subjects. The panel expressed concern about risks related to the Data and Safety Monitoring plan and recommended that adverse events be assessed at each participant encounter.

INCLUSION OF WOMEN PLAN: ACCEPTABLE

INCLUSION OF MINORITIES PLAN: ACCEPTABLE

INCLUSION OF CHILDREN PLAN: ACCEPTABLE

COMMITTEE BUDGET RECOMMENDATIONS: The budget was recommended as requested.

Footnotes for 1 R01 DK119478-01A1; PI Name: Ritchie, Natalie Dawn

NIH has modified its policy regarding the receipt of resubmissions (amended applications). See Guide Notice NOT-OD-14-074 at http://grants.nih.gov/grants/guide/notice-files/NOT-OD-14-074.html. The impact/priority score is calculated after discussion of an application by averaging the overall scores (1-9) given by all voting reviewers on the committee and multiplying by 10. The criterion scores are submitted prior to the meeting by the individual reviewers assigned to an application, and are not discussed specifically at the review meeting or calculated into the overall impact score. Some applications also receive a percentile ranking. For details on the review process, see http://grants.nih.gov/grants/peer_review_process.htm#scoring.

MEETING ROSTER

Dissemination and Implementation Research in Health Study Section
Healthcare Delivery and Methodologies Integrated Review Group
CENTER FOR SCIENTIFIC REVIEW
DIRH
10/10/2018 - 10/11/2018

Notice of NIH Policy to All Applicants: Meeting rosters are provided for information purposes only. Applicant investigators and institutional officials must not communicate directly with study section members about an application before or after the review. Failure to observe this policy will create a serious breach of integrity in the peer review process, and may lead to actions outlined in NOT-OD-14-073 at https://grants.nih.gov/grants/guide/notice-files/NOT-OD-14-073.html and NOT-OD-15-106 at https://grants.nih.gov/grants/guide/notice-files/NOT-OD-15-106.html, including removal of the application from immediate review.

CHAIRPERSON(S)

BARTELS, STEPHEN J, MD, MS
PROFESSOR AND DIRECTOR
THE MONGAN INSTITUTE
MASSACHUSETTS GENERAL HOSPITAL
BOSTON, MA 02114

MEMBERS

AALSMA, MATTHEW, PHD
PROFESSOR
DEPARTMENT OF PEDIATRICS
SCHOOL OF MEDICINE
INDIANA UNIVERSITY
INDIANAPOLIS, IN 46203

AMICO, KATHY RIVET, PHD *
ASSOCIATE PROFESSOR
DEPARTMENT OF HEALTH BEHAVIOR AND HEALTH EDUCATION
SCHOOL OF PUBLIC HEALTH
UNIVERSITY OF MICHIGAN
ANN ARBOR, MI 48109

BACKHUS, LEAH M, MD, MPH *
ASSOCIATE PROFESSOR
DEPARTMENT OF CARDIOTHORACIC SURGERY
STANFORD UNIVERSITY HOSPITAL
STANFORD, CA 94304

BAQUERO, BARBARA I, MPH, PHD *
ASSOCIATE PROFESSOR
DEPARTMENT OF COMMUNITY AND BEHAVIORAL HEALTH
COLLEGE OF PUBLIC HEALTH
THE UNIVERSITY OF IOWA
IOWA CITY, IA 52242

BECKER, SARA J, PHD *
ASSOCIATE PROFESSOR
CENTER FOR ALCOHOL AND ADDICTION STUDIES
SCHOOL OF PUBLIC HEALTH
BROWN UNIVERSITY
PROVIDENCE, RI 02912

BRANDT, HEATHER M, PHD *
ASSOCIATE PROFESSOR
DEPARTMENT OF HEALTH PROMOTION
EDUCATION AND BEHAVIOR
UNIVERSITY OF SOUTH CAROLINA
COLUMBIA, SC 29208

CARRASQUILLO, OLVEEN, MD, MPH
PROFESSOR OF MEDICINE AND PUBLIC HEALTH SCIENCES
CHIEF, DIVISION OF INTERNAL MEDICINE GERIATRICS
MILLER SCHOOL OF MEDICINE
UNIVERSITY OF MIAMI
MIAMI, FL 33101

CARSON, APRIL P, PHD
ASSOCIATE PROFESSOR
DEPARTMENT OF EPIDEMIOLOGY
UNIVERSITY OF ALABAMA AT BIRMINGHAM
BIRMINGHAM, AL 35294

CHARLEBOIS, EDWIN DUNCAN, MPH, PHD *
PROFESSOR
DEPARTMENT OF MEDICINE
SCHOOL OF MEDICINE
UNIVERSITY OF CALFORNIA, SAN FRANCISCO
SAN FRANCISCO, CA 94105

CLOUSE, KATE, PHD *
ASSISTANT PROFESSOR
VANDERBILT INSTITUTE FOR GLOBAL HEALTH
DIVISION OF INFECTIOUS DISEASES
DEPARTMENT OF MEDICINE
VANDERBILT UNIVERSITY MEDICAL CENTER
NASHVILLE , TN 37203

CORSO, PHAEDRA S, PHD
PROFESSOR
DEPARTMENT OF HEALTH POLICY AND MANAGEMENT
COLLEGE OF PUBLIC HEALTH
UNIVERSITY OF GEORGIA
ATHENS, GA 30602

DAVISON, KIRSTEN, PHD
ASSOCIATE PROFESSOR
DEPARTMENT OF NUTRITION
SCHOOL OF PUBLIC HEALTH
HARVARD T.H. CHAN
BOSTON, MA 02115

DOWNS, JENNIFER ALZOS, MD, PHD *
ASSISTANT PROFESSOR
DEPARTMENT OF MEDICINE
WEILL CORNELL MEDICAL COLLEGE
CORNELL UNIVERSITY
NEW YORK, NY 10065

DUBBERT, PATRICIA M, BSN, PHD *
PROFESSOR (RETIRED)
DEPARTMENT OF PSYCHIATRY
UNIVERSITY OF ARKANSAS FOR MEDICAL SCIENCES
LITTLE ROCK, AR 72205

EHRENTHAL, DEBORAH BETH, MD, MPH *
ASSOCIATE PROFESSOR
DIVISION OF REPRODUCTIVE AND POPULATION HEALTH
DEPARTMENT OF OBSTETRICS & GYNECOLOGY
UNIVERSITY OF WISCONSIN
SCHOOL OF MEDICINE AND PUBLIC HEALTH
MADISON, WI 53726

ELLIS, DEBORAH A, PHD *
PROFESSOR AND CHIEF OF BEHAVIORAL SCIENCES
DEPARTMENT OF FAMILY MEDICINE
WAYNE STATE UNIVERSITY
DETROIT, MI 48021

FIKS, ALEXANDER GABRIEL, MD *
ASSOCIATE PROFESSOR
DEPARTMENT OF PEDIATRICS
THE CHILDREN'S HOSPITAL OF PHILADELPHIA
PHILADELPHIA, PA 19104

GRIFFIN, BETH ANN, PHD
SENIOR STATISTICIAN, CO-DIRECTOR RAND
CENTER FOR CAUSAL INFERENCE
RAND CORPORATION
ARLINGTON, VA 22202

HAIDER, SADIA, MD, MPH *
ASSOCIATE PROFESSOR AND SECTION CHIEF
FAMILY PLANNING AND CONTRACEPTIVE RESEARCH
DEPARTMENT OF OBSTETRICS/GYNECOLOGY
UNIVERSITY OF CHICAGO
CHICAGO, IL 60637

HANNON, MARGARET, MPH, PHD
PROFESSOR
DEPARTMENT OF HEALTH SERVICES
UNIVERSITY OF WASHINGTON
SEATTLE, WA 98105

HOUSTON, THOMAS K II, MD, MPH
PROFESSOR AND DIVSION CHIEF
DEPARTMENT OF QUANTITATIVE HEALTH SCIENCES
DIVISION OF HEALTH INFORMATICS AND
IMPLEMENTATION SCIENCE; SCHOOL OF MEDICINE
UNIVERSITY OF MASSACHUSETTS
WORCESTER, MA 01655

HU, YE TONY, PHD *
ASSOCIATE PROFESSOR
SCHOOL OF BIOLOGICAL AND HEALTH
SYSTEMS ENGINEERING
ARIZONA STATE UNIVERSITY
TEMPE, AZ 85287-5001

HUDSON, SHAWNA V, PHD
PROFESSOR AND RESEARCH DIVISION CHIEF
DEPARTMENT OF FAMILY MEDICINE
AND COMMUNITY HEALTH
ROBERT WOOD JOHNSON MEDICAL SCHOOL
RUTGERS UNIVERSITY
NEW BRUNSWICK, NJ 08901

KLEINMAN, LAWRENCE C, MD *
DIRECTOR, CENTER FOR CHILD HEALTH & POLICY
DEPARTMENT OF PEDIATRICS
UH RAINBOW BABIES' AND CHILDREN'S HOSPITAL
SCHOOL OF MEDICINE
CASE WESTERN RESERVE UNIVERSITY
CLEVELAND, OH 44106

KUSHEL, MARGOT B, MD
PROFESSOR
DIVISION OF GENERAL INTERNAL MEDICINE
ZUCKERBERG SAN FRANCISCO GENERAL HOSPITAL
UNIVERSITY OF CALIFORNIA SAN FRANCISCO
SAN FRANCISCO, CA 94143

MILLER, CARLA K, PHD *
PROFESSOR
DEPARTMENT OF HUMAN SCIENCES
OHIO STATE UNIVERSITY
COLUMBUS, OH 43210

MILLER, DAVID P, MD, MS *
PROFESSOR
DEPARTMENT OF MEDICINE AND PUBLIC HEALTH
SCIENCES
WAKE FOREST SCHOOL OF MEDICINE
WINSTON-SALEM, NC 27157

MOISE, NATHALIE, MD *
ASSISTANT PROFESSOR OF MEDICINE
DIVISION OF GENERAL MEDICINE
COLUMBIA UNIVERSITY MEDICAL CENTER
NEW YORK, NY 10025

PALINKAS, LAWRENCE A, PHD
PROFESSOR AND CHAIR
DEPARTMENT OF CHILDREN, YOUTH AND FAMILIES
SUZZANE DWORAK-PECK SCHOOL OF SOCIAL WORK
UNIVERSITY OF SOUTHERN CALIFORNIA
LOS ANGELES, CA 90089

PARTHASARATHY, SAIRAM, MD *
PROFESSOR OF MEDICINE
DIVISION OF PULMONARY, ALLERGY, CRITICAL CARE
AND SLEEP MEDICINE
UNIVERSITY OF ARIZONA COLLEGE OF MEDICINE
TUCSON, AZ 85701

POLLINI, ROBIN A, PHD
ASSOCIATE PROFESSOR
DEPARTMENT OF BEHAVIORAL MEDICINE AND PSYCHIATRY
SCHOOL OF MEDICINE
WEST VIRGINIA UNIVERSITY
MORGANTOWN, WV 26506

RICHTER, KIMBER P, PHD *
PROFESSOR
DEPARTMENT OF PREVENTIVE MEDICINE
AND PUBLIC HEALTH
UNIVERSITY OF KANSAS MEDICAL CENTER
KANSAS CITY, KS 66160

SARPONG, DANIEL F, PHD
PROFESSOR OF BIOSTATISTICS, ENDOWED CHAIR AND
DIRECTOR
CENTER FOR MINORITY HEALTH AND HEALTH DISPARITIES
RESEARCH AND EDUCATION
COLLEGE OF PHARMACY
XAVIER UNIVERSITY
NEW ORLEANS, LA 70125

SHI, LIZHENG, MSPHARM, PHD *
REGENTS PROFESSOR
DEPARTMENT OF GLOBAL HEALTH MANAGEMENT AND
POLICY
TULANE UNIVERSITY SCHOOL OF PUBLIC HEALTH AND
TROPICAL MEDICINE
NEW ORLEANS, LA 70112

SPAULDING, AARON, PHD *
ASSOCIATE CONSULTANT
DEPARTMENT OF HEALTH SCIENCES RESEARCH
DIVISION OF HEALTH CARE POLICY AND RESEARCH
MAYO CLINIC ROBERT D AND PATRICIA E KERN
CENTER FOR THE SCIENCE OF HEALTH CARE DELIVERY
JACKSONVILLE, FL 32224

STOUTENBERG, MARK, MSPH, PHD *
ASSOCIATE PROFESSOR
HEALTH AND HUMAN PERFORMANCE
UNIVERSITY OF TENNESSEE AT CHATTANOOGA
CHATTANOOGA, TN 37403

SVENSSON, CRAIG K, PHD, PHMD *
DEAN EMERITUS AND PROFESSOR
COLLEGE OF PHARMACY
PURDUE UNIVERSITY
WEST LAFAYETTE, IN 47907

TUCKER, JOSEPH DAVID, MD, PHD *
ASSOCIATE PROFESSOR AND DIRECTOR
CENTER FOR INFECTIOUS DISEASES
UNC PROJECT-CHINA
UNIVERSITY OF NORTH CAROLINA SCHOOL OF MEDICINE
CHAPEL HILL, NC 27599

WHEELER, DARRELL P, PHD *
PROVOST AND SENIOR VICE PRESIDENT FOR ACADEMIC
AFFAIRS
IONA COLLEGE
NEW ROCHELLE, NY 10801

WILLIAMS, LOVORIA, PHD *
ASSOCIATE PROFESSOR
COLLEGE OF NURSING
UNIVERSITY OF KENTUCKY
LEXINGTON, KY 40536

ZHANG, QI, MD, PHD *
ASSOCIATE PROFESSOR
SCHOOL OF COMMUNITY AND ENVIRONMENTAL HEALTH
OLD DOMINION UNIVERSITY
NORFOLK, VA 23529

MAIL REVIEWER(S)

LEVY, BARCEY THURSTON, MD, PHD
PROFESSOR
DEPARTMENT OF FAMILY MEDICINE
UNIVERSITY OF IOWA
IOWA CITY, IA 52242-1009

SCIENTIFIC REVIEW OFFICER

FERGUSON, YVONNE OWENS, PHD
SCIENTIFIC REVIEW OFFICER
CENTER FOR SCIENTIFIC REVIEW
NATIONAL INSTITUTES OF HEALTH
BETHESDA, MD 20892

EXTRAMURAL SUPPORT ASSISTANT

JONES, BELINDA
EXTRAMURAL SUPPORT ASSISTANT
CENTER FOR SCIENTIFIC REVIEW
NATIONAL INSTITUTES OF HEALTH
BETHESDA, MD 20892

* Temporary Member. For grant applications, temporary members may participate in the entire meeting or may review only selected applications as needed.

Consultants are required to absent themselves from the room during the review of any application if their presence would constitute or appear to constitute a conflict of interest.
